# Label-Free Quantitative Proteomics Analysis in Susceptible and Resistant *Brassica napus* Cultivars Infected with *Xanthomonas campestris* pv. *campestris*

**DOI:** 10.3390/microorganisms9020253

**Published:** 2021-01-27

**Authors:** Md Tabibul Islam, Bok-Rye Lee, Van Hien La, Dong-Won Bae, Woo-Jin Jung, Tae-Hwan Kim

**Affiliations:** 1 Department of Animal Science, Institute of Agricultural Science and Technology, College of Agriculture & Life Science, Chonnam National University, Gwangju 61186, Korea; tabibul@vt.edu (M.T.I.); turfphy@jnu.ac.kr (B.-R.L.); hiencnsh88@gmail.com (V.H.L.); 2 Alson H. Smith Jr. Agricultural Research and Extension Center, School of Plant and Environmental Sciences, Virginia Tech, Winchester, VA 22602, USA; 3 Asian Pear Research Institute, Chonnam National University, Gwangju 61186, Korea; 4 Central Instrument Facility, Gyeongsang National University, Jinju 52828, Korea; bdwon@gnu.ac.kr; 5 Division of Applied Bioscience and Biotechnology, Institute of Environmentally Friendly Agriculture (IEFA), College of Agriculture and Life Science, Chonnam National University, Gwangju 61186, Korea; woojung@chonnam.ac.kr

**Keywords:** *Brassica napus*, proteolysis, photosynthesis-related proteins, redoxins, redox status, *Xanthomonas campestris* pv. *campestris*

## Abstract

Black rot, caused by *Xanthomonas campestris* pv. *campestris* (*Xcc*), is the main disease of cruciferous vegetables. To characterize the resistance mechanism in the *Brassica napus*–*Xcc* pathosystem, *Xcc*-responsive proteins in susceptible (cv. Mosa) and resistant (cv. Capitol) cultivars were investigated using gel-free quantitative proteomics and analysis of gene expression. This allowed us to identify 158 and 163 differentially expressed proteins following *Xcc* infection in cv. Mosa and cv. Capitol, respectively, and to classify them into five major categories including antioxidative systems, proteolysis, photosynthesis, redox, and innate immunity. All proteins involved in protein degradation such as the protease complex, proteasome subunits, and ATP-dependent Clp protease proteolytic subunits, were upregulated only in cv. Mosa, in which higher hydrogen peroxide accumulation concurred with upregulated superoxide dismutase. In cv. Capitol, photosystem II (PS II)-related proteins were downregulated (excepting PS II 22 kDa), whereas the PS I proteins, ATP synthase, and ferredoxin-NADP^+^ reductase, were upregulated. For redox-related proteins, upregulation of thioredoxin, 2-cys peroxiredoxin, and glutathione S-transferase occurred in cv. Capitol, consistent with higher NADH-, ascorbate-, and glutathione-based reducing potential, whereas the proteins involved in the C_2_ oxidative cycle and glycolysis were highly activated in cv. Mosa. Most innate immunity-related proteins, including zinc finger domain (ZFD)-containing protein, glycine-rich RNA-binding protein (GRP) and mitochondrial outer membrane porin, were highly enhanced in cv. Capitol, concomitant with enhanced expression of *ZFD* and *GRP* genes. Distinguishable differences in the protein profile between the two cultivars deserves higher importance for breeding programs and understanding of disease resistance in the *B. napus*–*Xcc* pathosystem.

## 1. Introduction

Oilseed rape (*Brassica napus*) is an important agro-economic crop of the *Brassicaceae* family grown worldwide for vegetable oil, animal feeds, and as an alternative fuel. *Xanthomonas campestris* pv. *campestris* (*Xcc*), a Gram-negative hemibiotrophic bacterial pathogen, is the causal agent of black rot disease, which has become a major threat to the quality and production of cruciferous vegetables around the world [1,2]. As an important strategy of black rot control, development of black rot-resistant cultivars has long been considered. Several studies have attempted to identify the sources of resistance towards *Xcc* in *Brassica* species [3]. Despite a number of genetic studies on resistance mechanisms, so far, the available sources of resistance to *Xcc* are limited [1]. Over recent decades, the roles of secondary metabolites in plant disease resistance systems have been evaluated to better understand the interaction between the host plant and *Xcc*. Metabolites of the phenylpropanoid pathway such as hydroxycinnamic acid amides, phenolic glucosides, and flavonoids, were shown to be involved in resistance in several pathosystems [4,5,6]. Previously, our group has also reported that the resistant interaction is characterized by an accumulation of flavonoids and hydroxycinnamic acids, with an enhancement of phenylpropanoid synthesis pathway-related genes in the *B. napus–Xcc* pathosystem [2].

The characterization of plant–pathogen interactions, including the pathogen-induced diverse signaling and biochemical metabolic pathways [7], would be an efficient approach to better understand the complex resistance mechanisms. Pathogen-responsive alteration of transcriptomes has been documented in different pathosystems [8]. However, changes in transcription often do not correspond to changes in protein expression [9]. Thus, a comparative proteomic analysis would be an efficient and powerful approach to screen differentially expressed proteins in relation to susceptible and/or resistant interactions. Previously, comparative proteomics between susceptible and resistant interactions has been employed to better understand defense mechanisms in different pathosystems [10,11]. Accumulation of redox-related proteins (oxidoreductase and peroxidase) and other defensive proteins was induced during the resistant interaction in the Arabidopsis–*P. syringe* pv. *tomato* pathosystem [10]. The downregulation of proteins related to photosynthesis, energy, and defense was associated with the susceptible *B. oleracea–Xcc* interaction [11]. However, traditional gel-based proteomics, which is biased towards hydrophilic proteins with higher abundance, is not sufficient for higher coverage and more accurate quantification [12]. High-throughput quantitative proteomics studies using mass spectrometry-based techniques [9] have emerged to figure out the distinct biochemical and metabolic signatures. Thus, in this study, we employed label-free and liquid chromatography–tandem mass spectrometry (LC–MS/MS)-based quantitative proteomics to reveal the global proteomic responses in two *B. napus* cultivars with contrasting disease susceptibility responses to black rot (cv. Capitol as resistant and cv. Mosa as susceptible cultivars) [2]. To the best of our knowledge, this is the first study to use high-throughput label-free quantitative proteomics to characterize susceptible and resistant interactions in the *B. napus–Xcc* pathosystem.

In this study, we hypothesized that the *Xcc* infection-responsive protein profile would be unique in different cultivars; therefore, a direct comparison of differentially expressed proteins would allow us to characterize the defense mechanisms against black rot disease. To test this hypothesis, the proteins responsive to *Xcc* infection were screened in susceptible and resistant cultivars, and functionally classified. The functionally classified differentially expressed proteins associated with resistance were interpreted with the analyzed data of physiological parameters and the expression of genes.

## 2. Materials and Methods

### 2.1. Plant Growth and Pathogen Stress Treatment

Surface-sterilized seeds of two oilseed rape (*B. napus*) cultivars (cv. Capitol and cv. Mosa) were grown in pots (1.65 L). When the seedlings had grown up to the six-leaf stage, they were divided into two groups—i.e., control (noninoculated) and another with pathogen inoculation. The pathogenic bacterial strain (*Xanthomonas campestris* pv. *campestris*) was obtained from the Korean Agricultural Culture Collection. Bacterial inoculum was cultured on Yeast Dextrose Calcium Carbonate (YDC) agar plates for 48 h at 30 °C, after which the bacterial colonies were scraped from plates and adjusted to a concentration of 10^8^ cfu/mL (0.2 O.D., A_600_ nm) with 0.85% NaCl solution. The experiment was conducted with a completely randomized design with three biological replications. Three different pots (e.g., a single plant per pot) were allocated for each treatment. Six fully expanded leaves of one group were inoculated with Xcc inoculum by the clipping of the leaf edges near the veins using mouth tooth forceps, whereas those of another group were clipped with water. The inoculation process was followed by clipping off the leaf edges near the veins using mouth-toothed forceps. For every inoculation, the forceps were dipped into the bacterial suspension. Fourteen days after inoculation, six leaves of each plant were sampled from both pathogen-inoculated or control (noninoculated) plants in the same leaf rank order. Six combined leaves were considered as a biological replicate of each treatment. The leaves were collected from the plants for the proteomics analysis and evaluation of different biochemical attributes; they were immediately frozen in liquid nitrogen and stored in a deep freezer (−80 °C) for further analysis.

### 2.2. Determination of Reactive Oxygen Species (ROS) Content

The hydrogen peroxide (H_2_O_2_) levels were measured as described by Islam et al. [2]. The extracted solution was mixed with 0.1% titanium chloride in 20% (*v*/*v*) H_2_SO_4_, and the mixture was centrifuged at 10,000× *g* for 5 min. The absorbance of the supernatant was measured at 410 nm. H_2_O_2_ concentration was calculated using the extinction coefficient of 0.28 mmol^−1^·cm^−1^.

### 2.3. Protein Extraction and Quantification

Total proteins were extracted according to a modified tricholoroacetic acid (TCA)/acetone/phenol precipitation method [13]. Leaf tissues were homogenized to a fine powder in cold TCA/acetone buffer composed of 10% TCA and 0.07% 2-mercaptoethanol in acetone. The pellet was washed twice with cold acetone and then air-dried. Equal volumes of SDS extraction buffer (30% sucrose, 1–2% SDS, 0.1 M Tris-HCl, pH 8.8) and saturated phenol (pH 8.0) were added and mixed vigorously, then centrifuged at 12,000× *g* for 15 min at 4 °C. The phenol phase was transferred to a new tube and 0.1 M ammonium acetate in methanol (five times the volume of the phenol phase) was added. Proteins were suspended in the lysis buffer composed of 7 M urea, 2 M thiourea, 4% CHAPS, 1 mM PMSF, 50 mM DTT, 0.5% IPG buffer (GE Healthcare, Bi-Science Corp, Las Vegas, NV, USA). Protein concentrations were determined by using the 2D Quant kit (GE Healthcare, Bi-Science Corp). The resultant samples were stored at −80 °C before analysis.

### 2.4. Protein Digestion

Protein samples were digested using a digestion buffer, which contained 25 mM ammonium bicarbonate, 0.1% *N*-octyl glucoside, and 50 ng/mL of sequencing grade trypsin (Promega, Madison, MI, USA) for rehydration. After rehydration, the samples were incubated overnight at 37 °C in digestion buffer (without trypsin) to allow enzymatic cleavage in the siliconized tube. Peptides were extracted with 66% acetonitrile, 33% water, 0.1% trifluoroacetic acid (TFA). After centrifugation, the peptides were transferred to a new tube and dried with a speedvac (Hanil, Seoul, Korea). The dried peptides from the gel slices were stored at −80 °C before analysis.

### 2.5. LC–MS/MS and Data Analysis

For gel-free proteomic analysis, protein samples were resuspended in formic acid solution (0.1% formic acid in water). Online NanoHPLC was conducted on an Ekisigent nanoLC415 system (EKsigent, Dublin, CA, USA). Each peptide was transferred to an analytical ChromXP C18 column (75 µm × 15 cm, 3 µm, 120 Å), and eluted at a flow rate of 300 nL/min using a 90 min gradient with analysis solvent (0.1% formic acid in water). Data acquisition was performed with a triple time-of-flight (TOF) 6600 system (SCIEX, Redwood City, CA, USA) coupled with a nanospray source (New Objective, Woburn, MA, USA), with a pulled 10 µm fused silica emitter, 360/20 µm (New Objective, Woburn, MA, USA). Data were acquired using an ion spray voltage floating (ISVF) of 2.3 kV, curtain gas of 28 psi, ion source gas (GSI) of 15 psi, and interface heater temperature of 150 °C. For information-dependent acquisition (IDA), the range of survey scans was set between 250 and 2000 *m*/*z* (250 ms accumulation time) followed by a dependent MS/MS scan with a mass range set between 100 and 2000 *m*/*z* (100 ms accumulation time). After MS/MS analysis, data files were processed using UniProt and Protein Pilot 5.0.1 (SCIEX, Redwood City, CA, USA) database software. Based on the combined MS and MS/MS spectra, proteins were successfully identified at a 95% or higher confidence interval, using their scores in the MASCOT v2.5 search engine (Matrix Science Ltd., London, UK) and the following search parameters: *Brassica* database, trypsin as the digestion enzyme, single missed cleavage site, fixed modifications of carbamidomethyl (C) and oxidation of methionine, ±0.1 Da precursor ion tolerance, and ± 0.1 Da MS/MS fragment ion tolerance.

### 2.6. Identification of Differentially Accumulated Proteins (DAPs)

Protein identification was undertaken using a thorough search effort using ProteinPilot 5.0.1 software with the Paragon algorithm. The search parameters were defined as iodoacetamide modified for cysteine alkylation, and trypsin as the digestion enzyme. Tandem mass spectrometry data were searched against a database comprising Uniprot-*Brassica* (version 2107/08) and *Brassica* peptide sequences (sequence downloaded in August 2017; 209326 sequences). The database search results were manually curated to yield the protein identifications using 1% global false discovery rate (FDR), determined by the in-built FDR tool within ProteinPilot software. Scaffold (version Scaffold_4.8.4, Proteome Software Inc., Portland, OR, USA) was used to validate MS/MS-based peptides and protein identifications. Peptide identifications were accepted if they could be established at greater than 95.0% probability, to achieve an FDR less than 1% by the Scaffold Local FDR algorithm. The fold changes of ≥1.5 and ≤0.5 and *p*-value of < 0.05 (paired *t*-test) were considered as significant upregulated or downregulated differentially accumulated proteins, respectively. 

### 2.7. Functional Classification

Functional annotation of DAPs was performed using Blast2GO (https://www.blast2go.com). The peptide sequences of all DAPs were extracted and submitted to NCBI for BLAST search (http://blast.ncbi.nlm.nih.gov/Blast.cgi) using default parameters, with *B. napus* as the organism filter. The BLAST results were downloaded as XML files and manually inputted into Blast2GO for gene ontology (GO) mapping.

### 2.8. Protease Activity In-Gel Staining

Total proteolytic activity of the leaf extract was determined using the method by Beyene et al. [14] using SDS-PAGE. Visualization of proteases, after electrophoretic separation in 12.5% SDS-PAGE-containing gelatin, was performed according to Beyene et al. [14].

### 2.9. Glutathione and Ascorbate Redox Status

Total (tGSH) and oxidized (GSSG) glutathione were analyzed using a commercial kit (Prod. No. GT40, Oxford Biomedical Research, Oxford, UK). Ascorbate (AsA) and dehydroascorbate (DHA) contents were determined according to Kampfenkel et al. [15]. The assay is based on the reduction of Fe^3+^ to Fe^2+^ by AsA and the spectrophotometric detection of Fe^2+^ complexed with 2,2′-dipyridyl at 525 nm. DHA was reduced to AsA by preincubating the sample with dithiothreitol (DTT). Excess DTT was removed with *N*-ethylmaleimide (NEM), and total AsA was determined by the 2,2′-dipyridyl method. Determination of oxidized and reduced pyridine nucleotide content was conducted as described by Noctor and Queval [16]. For extraction of NAD^+^ and NADH, 200 mg fresh leaves were homogenized with 0.8 mL of 0.2 N HCl (for NAD^+^) and 0.2 M NaOH (for NADH). Following this, 100 µL extract was heated at 95 °C for 1 min and, later, cooled in an ice bath. For the NAD^+^ assay, the supernatant was neutralized by 0.2 M NaOH to a final pH of 5–6, and NADH was neutralized by 0.2 N HCl to a final pH of 7–8. Forty microliters of this solution was added to the reaction mixture containing 0.1 M HEPES (pH 7.5) which consisted of 2 mM Na_2_EDTA, 1.2 mM dichlorophenolindophenol (DCPIP), 20 mM phenazine methosulfate (PMS), and 15 μL absolute ethanol. The reaction started by adding 10 μL alcohol dehydrogenase (ADH, 2500 U) for NADH/NAD^+^ measurement. The content of NAD^+^ and NADH were determined by the standard curve with a concentration range of 1–100 pmol.

### 2.10. Isolation of Total RNA and Quantitative Real-Time PCR

Total RNA was isolated from 200 mg leaf tissue using the RNAiso total RNA isolation system (TaKaRa). First-strand cDNAs were synthesized using the GoScript Reverse Transcription System (TaKaRa). The gene expression level was quantified on a light cycler real-time PCR detection system (Bio-Rad) with SYBR Premix Ex TaqTM (TaKaRa, Dalian, China). The primer sequences are presented in Supplementary Table S3. All the quantifications were normalized to actin. The qRT-PCR reactions were performed in triplicate for each of the three independent samples. Quantification of the relative transcript level was performed using the 2^−∆∆*C*t^ method [17].

### 2.11. Statistical Analysis

A completely randomized design was used with three replicates for each treatment. Duncan’s multiple range test was employed to compare the means of separate replicates. Statistical significance was *p* < 0.05. Statistical analysis of all measurements was carried out using the software SAS 9.1.3 (SAS Institute Inc., Cary, NC, USA).

## 3. Results

### 3.1. Disease Symptoms and H_2_O_2_ Accumulation in Response to Xcc

**Figure 1:**
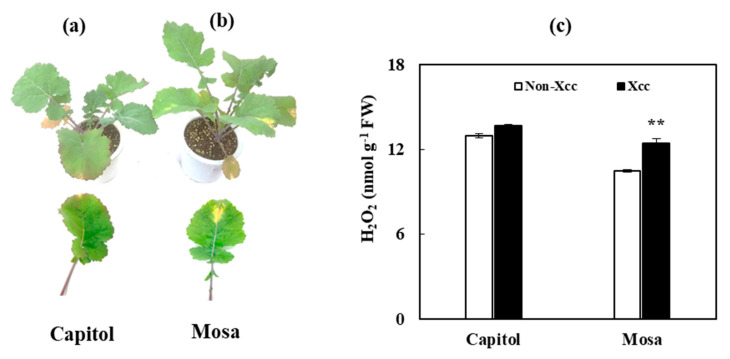
V-shaped necrotic lesion in the leaf of *B. napus* cultivars (**a**) cv. Capitol, (**b**) cv. Mosa and (**c**) H_2_O_2_ content in response Table S3. Asterisks indicate significant differences between the noninoculated *Xanthomonas campestris* pv. *campestris* (Xcc) (control) and Xcc-inoculated plants; ** *p* < 0.01.

V-shaped necrosis on leaf margins are the common symptoms of this devastating disease. Following *Xcc* inoculation, severe V-shaped necrotic areas occurred in cv Mosa (Figure 1b), along with H_2_O_2_ accumulation which was significantly higher than that in the non-*Xcc*-inoculated plants (Figure 1c), and cv Capitol was almost symptomless (Figure 1a).

### 3.2. Overview of Quantitative Proteomics

**Figure 2:**
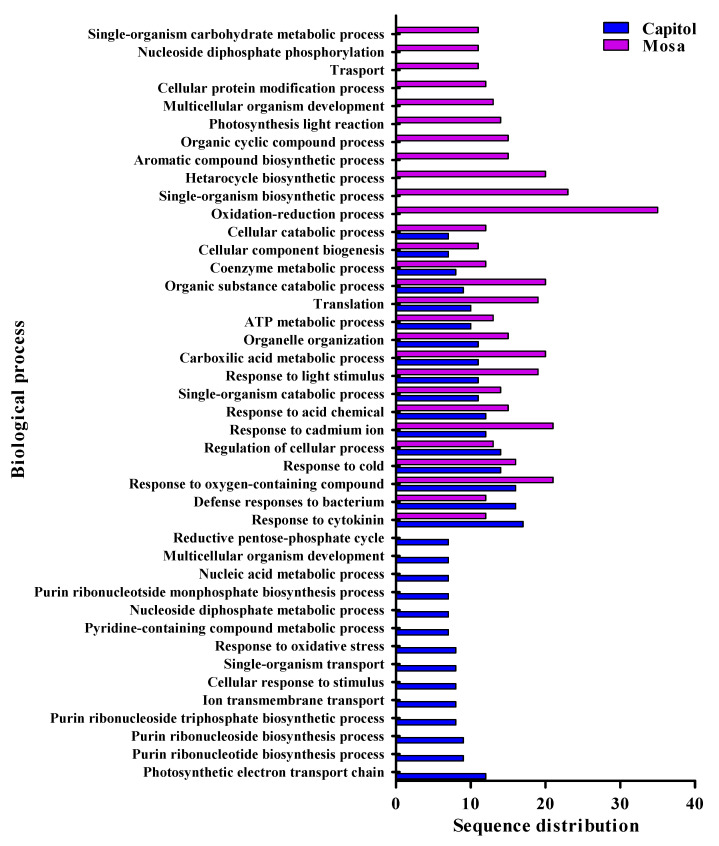
Functional classification of *B. napus* resistant cultivar (cv. Capitol) and susceptible cultivar (cv. Mosa) proteins in responses to *Xanthomonas campestris* pv. *campestris* (*Xcc*). Proteins were classified by gene ontology analysis with BLAST2GO on the basis of their biological processes.

A total of 1520 proteins were obtained from the label-free LC–MS/MS proteomic analysis of *Xcc*-infected or healthy leaves in two *B. napus* cultivars with contrasting susceptibility to *Xcc*. It was revealed that 163 and 158 proteins were differentially expressed following *Xcc* infection of cv. Capitol and cv. Mosa, respectively (Figure S1). Of 321 differentially expressed proteins in two *B. napus* cultivars with contrasting susceptibility to *Xcc*, 198 were successfully annotated with gene ontology (GO) terms using Blast2GO (Tables S1 and S2). These were then sorted into different biological processes (Figure 2), including regulation of cellular process, metabolic process, defense responses to bacterium, responses to stimuli, electron transport, and major cellular component which includes chloroplast envelope, thylakoid membrane, apoplast, plasma membrane (Figure S2), as well as major molecular functions (Figure S3) such as protein binding, oxidoreductase activity, RNA binding, ATP binding, hydrolase activity, and transferase activity.

### 3.3. Differentially Expressed Proteins in Response to Xcc

Based on the V-shaped necrotic lesions of leaves at 14 days postinoculation (DPI) of the resistant (cv. Capitol) and susceptible (cv. Mosa) cultivars of *B. napus* infected with *Xcc*, label-free global proteomics analysis was performed. From this analysis, 74 and 124 differentially expressed proteins were functionally classified for the resistant and susceptible cultivars, respectively. Compared with the non-*Xcc* inoculated plants, in *Xcc*-inoculated plants, 42 and 47 proteins were significantly upregulated (fold change ≥1.5 and *p*-value of < 0.05) in resistant (cv. Capitol) and susceptible (cv. Mosa) cultivars, respectively, compared to respective non-*Xcc* inoculated plants. The functionally classified proteins cover a wide range of cellular components, molecular functions, and biological processes. In the resistance interaction (between cv. Capitol and *Xcc*), there were several proteins involved in different biological processes, including defense responses to bacterium (21.6% of total proteins), proteins responsive to oxidative stress (10.2%), response to cytokinin (22.9%), proteins responsive to different stimuli (cold, light, cadmium ion, acid chemicals, 77.1%), photosynthetic electron transport chain (16.2%), and proteins involved in purine-pyrimidine metabolism (54.1%) (Figure 2). In contrast, in the susceptible interaction (between cv. Mosa and *Xcc*), proteins were identified that are involved in defense response to bacterium (9.6%), oxidation-reduction processes (28.2%), responses to different stimuli (cold, light, cadmium ion, acid chemicals) (57.2%), responses to cytokinin (9.6%), and photosynthesis (11.3%) (Figure 2).

### 3.4. Proteolysis-Related Proteins

**Figure 3:**
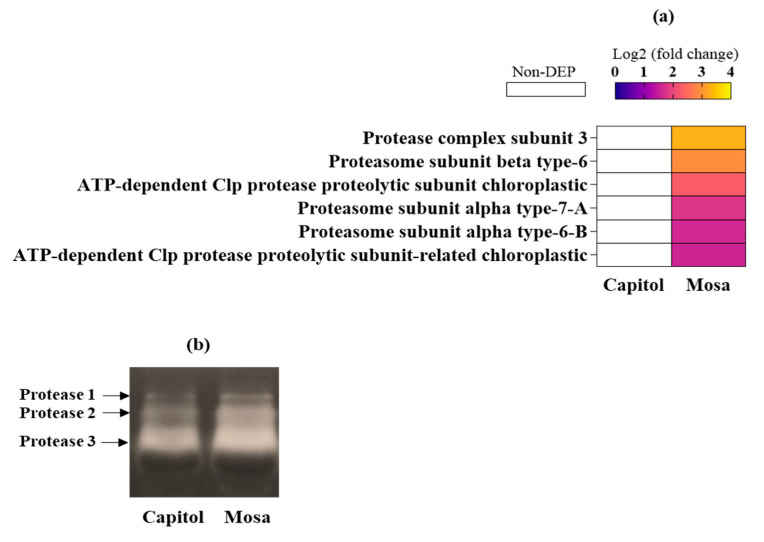
Differentially accumulated proteins involved in proteolysis in *Brassica. napus* cultivars (cv. Mosa) in responses to *Xanthomonas campestris* pv. *campestris* (Xcc). (**a**) Significantly expressed proteins (fold changes of ≥ 1.5 and ≤ 0.5 with *p* < 0.05, upregulated and downregulated, respectively) are presented as Log2 fold changes (Xcc/Non-Xcc). DEP—differentially expressed protein; (**b**) in-gel staining of protease activity in resistant cultivar (cv. Capitol) and (**b**) susceptible cultivar (cv. Mosa) proteins in responses to *Xanthomonas campestris* pv. *campestris* (Xcc).

Proteins involved in proteolysis, including different subunits of proteasome and Clp protease also significantly accumulated in the susceptible cultivar (cv. Mosa) in response to *Xcc* (Figure 3a). The protease active staining gel validated the obtained results of label free proteomics assay, as shown the presence of the brighter bands for the cv. Mosa than for the cv. Capitol in response to *Xcc* inoculation (Figure 3b).

### 3.5. Photosynthesis-Related Proteins

**Figure 4:**
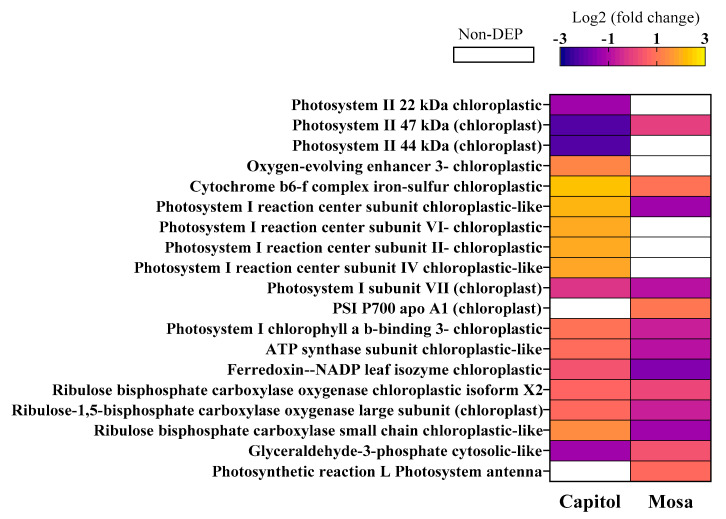
Profile of the photosynthesis-related differentially accumulated proteins in *B. napus* cultivars in responses to *Xanthomonas campestris* pv. *campestris* (Xcc). Significantly expressed proteins (fold changes of ≥ 1.5 and ≤ 0.5 with *p* < 0.05, upregulated and downregulated, respectively) are presented as Log2 fold changes (Xcc/Non-Xcc). DEP—differentially expressed protein.

A distinct alteration of the photosynthesis-related proteins occurred in resistant (cv. Capitol) and susceptible (cv. Mosa) cultivars in response to *Xcc* infection. In cv. Mosa, photosystem II (PS II) proteins, including PS II 22 kDa, were highly expressed; however, all the PS II-related proteins (22, 47 and 44 kDa) were remarkably repressed in cv. Capitol (Figure 4). In response to *Xcc*, the PS I reaction center protein and PS I chlorophyll a/b-binding 3 were significantly expressed in the resistant cultivar (cv. Capitol), along with the oxygen-evolving enhancer protein, cytochrome b_6_f complex, photosystem I (PS I)-related proteins, and ATP synthase. In addition, Calvin cycle-related proteins were also significantly enhanced in cv. Capitol (Figure 4).

### 3.6. ROS Production and Redox-Related Proteins

**Figure 5:**
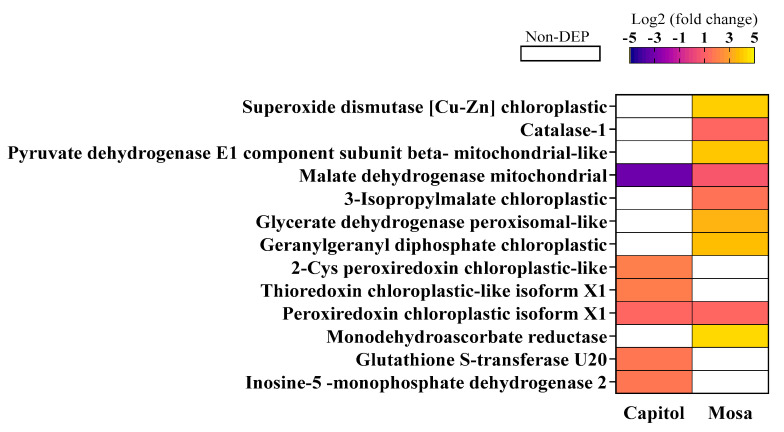
Reactive oxygen species (ROS) and redox-related differentially accumulated proteins in *B. napus* cultivars in response to *Xanthomonas campestris* pv. *campestris* (*Xcc*). Significantly expressed proteins (fold changes of ≥ 1.5 and ≤ 0.5 with *p* < 0.05, upregulated and downregulated, respectively) are presented as Log2 fold changes (*Xcc*/Non-*Xcc*). DEP—differentially expressed protein.

Superoxide dismutase (SOD) and catalase (CAT) are two major enzymes which are involved in ROS scavenging and/or generation; both these proteins were upregulated in cv. Mosa following *Xcc* inoculation, and the accumulation of SOD was much higher than CAT (Figure 5). The citric acid cycle-related proteins mitochondrial pyruvate dehydrogenase and malate dehydrogenase were upregulated in the susceptible cultivar, whereas malate dehydrogenase was downregulated in the resistant cultivar (cv. Capitol) in *Xcc*-inoculated plants (Figure 5). Moreover, 2-cys peroxiredoxin, peroxiredoxin, and thioredoxin are important redox-related proteins that maintain the redox homeostasis. In the resistant cultivar (cv. Capitol), following *Xcc* inoculation, 2-cys peroxiredoxin and thioredoxin accumulated significantly, compared to controls (Figure 5).

### 3.7. Immune Response Protein Accumulation in Response to Xcc

**Figure 6:**
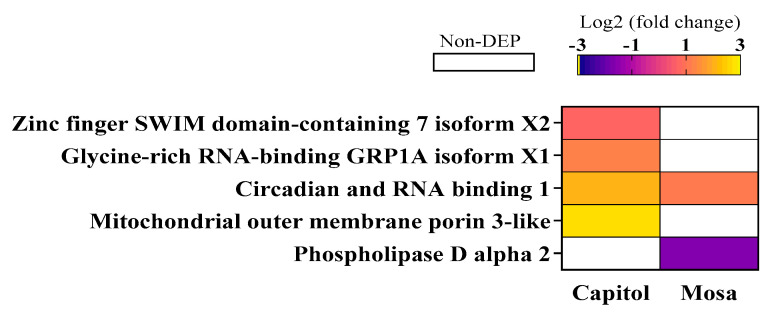
Differentially accumulated proteins involved in the plant innate immune responses in *B. napus* cultivars in response to *Xanthomonas campestris* pv. *campestris* (Xcc). Significantly expressed proteins (fold changes of ≥ 1.5 and ≤ 0.5 with *p* < 0.05, upregulated and downregulated, respectively) are presented as Log2 fold changes (Xcc/Non-Xcc). DEP—differentially expressed protein.

Among the identified proteins in the resistant cultivar cv. Capitol, three proteins are involved in immune responses, including zinc finger SWIM domain-containing 7 isoform X2 (ZFD), glycine-rich RNA-binding GRP1A isoform X1, and mitochondrial outer membrane porin; these were significantly upregulated in the resistant cultivar cv. Capitol in response to *Xcc*. However, in the susceptible cultivar cv. Mosa following *Xcc* inoculation, phospholipase D alpha 2, which is a secondary signaling protein in plant innate immune responses, was significantly repressed (Figure 6).

### 3.8. NADH, Ascorbate, and Glutathione Redox Status

**Figure 7:**
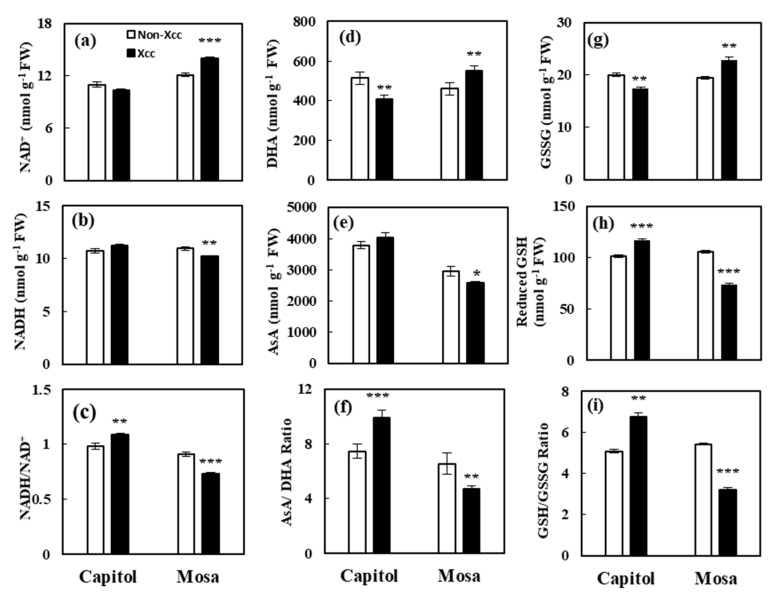
NADH, ascorbate, and glutathione redox statuses in responses to *Xanthomonas campestris* pv. *campestris* (Xcc) inoculation in *B. napus* cultivars: (**a**) NAD^+^, (**b**) NADH, (**c**) NADH/NAD^+^ ratio, (**d**) dehydroascorbate (DHA), (**e**) reduced ascorbate (AsA), and (**f**) the ratio of AsA to DHA, (**g**) oxidized glutathione (GSSG), (**h**) reduced glutathione (GSH) content, and (**i**) the ratio of GSH to GSSG. Data are presented as means ± SE for *n* = 3. Asterisks indicate significant differences between the Xcc noninoculated (control) and Xcc-inoculated plants; * *p* < 0.05, ** *p* < 0.01, and *** *p* < 0.001.

*Xcc* inoculation altered the NADH-, glutathione-, and ascorbate-based redox statuses in both resistant and susceptible cultivars (Figure 7). NAD^+^ (*p* < 0.001), dehydroascorbate (DHA; *p* < 0.01), and oxidized glutathione (GSSG; *p* < 0.01) contents were significantly increased by *Xcc* inoculation in the susceptible cultivar cv. Mosa (Figure 7a,d,g). The reduced form of glutathione was markedly enhanced in the *Xcc*-inoculated resistant cultivar (cv. Capitol) compared to non-*Xcc*-inoculated plants (Figure 7h). The resulting NADH/NAD^+^, AsA/DHA, and GSH/GSSG ratios significantly increased in the resistant cultivar (cv. Capitol), whereas these ratios decreased significantly in the susceptible cultivar (cv. Mosa) in response to *Xcc* infection (Figure 7c,f,i).

### 3.9. Expression of Genes

**Figure 8:**
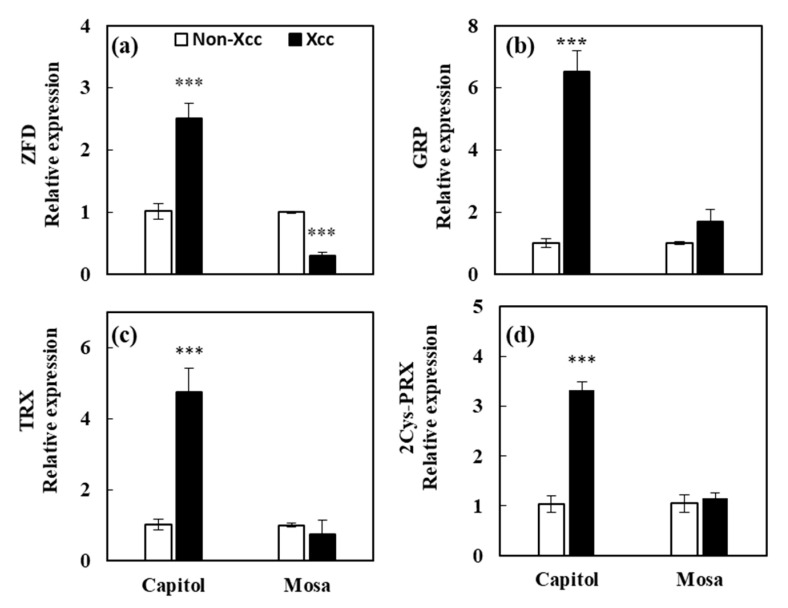
Relative expression of genes (**a**) zinc finger SWIM domain-containing 7 isoform X2 (ZFD), (**b**) glycine-rich RNA-binding GRP1A isoform X1 (GRP), (**c**) thioredoxin (TRX), (**d**) 2-cys peroxiredoxin (2Cys-PRX) in control (open bar) and *Xanthomonas campestris* pv. *campestris* (Xcc)-inoculated (filled bar) leaves of two different *B. napus* cultivars as affected by Xcc inoculation. Asterisks indicate significant differences between the Xcc noninoculated (control) and Xcc-inoculated plants; *** *p* < 0.001.

The relative expression of immune response and redoxregulating genes were upregulated in the resistant cultivar (cv. Capitol), including zinc finger SWIM domain-containing 7 isoform X2 (*ZFD*, + 147.1%; Figure 8a), glycine-rich RNA-binding GRP1A isoform X1 (*GRP*, + 538.3%; Figure 8b), thioredoxin (*TRX*, + 364.3%; Figure 8c), and 2-cys peroxiredoxin (*2 Cys-PRX*, + 222.3%; Figure 8d), whereas *ZFD* was repressed (−70.1%) in the susceptible cultivar (cv. Mosa) in response to *Xcc* inoculation (Figure 8a).

## 4. Discussion

The bacterial plant pathogen *Xanthomonas campestris* pv. *campestris* (*Xcc*) is a causal agent of black rot in most cruciferous plants. The common symptoms are chlorosis and V-shaped necrosis on the leaf [2]. In this study, at 14 days after *Xcc* inoculation, the disease symptoms were visibly developed. It was revealed that cv. Mosa is susceptible to *Xcc*, as the cultivar developed more severe V-shaped necrosis and had significant accumulation of H_2_O_2_, whereas cv. Capitol was resistant (Figure 1). In our previous study [2], an accumulation of defensive metabolites with enhanced expressions of genes involved in the biosynthesis of flavonoids (*CHS*), proanthocyanidins (*ANR*), and hydroxycinnamic acids (*F5H*), and a higher redox status were observed in the resistant cultivar (cv. Capitol), whereas the opposite results were obtained in the susceptible cultivar (cv. Mosa). In this context, a high-throughput gel-free quantitative proteomics analysis was performed to determine the metabolic and molecular fundamentals of the resistant and/or susceptible interactions in the *B. napus–Xcc* pathosystem.

### 4.1. ROS Production and Proteolysis

Reactive oxygen species (ROS) accumulation is one of the earliest responses of plant cells following exposure to various abiotic and biotic stresses [2,18]. The enzyme NADPH-dependent oxidase located in the plasma membrane produces superoxide (O_2_^.−^) by transferring electrons from NADPH to the incompletely reduced oxygen under stress conditions. The dismutation of O_2_^.−^ is catalyzed by superoxide dismutase (SOD) to produce H_2_O_2_, which is in turn converted to O_2_ and H_2_O by catalase (CAT) [19]. In the cv. Mosa, *Xcc* infection induced a downregulation of the proteins involved in electron transfer in the Calvin cycle (Rubisco large and small subunits, Figure 4), leading to insufficient production of the electron acceptor (e.g., NADP^+^). This might be partly responsible for higher ROS accumulation during a susceptible interaction (Figure 1c), because photosynthetic cells produce more superoxide when levels of NADP^+^ are not sufficient for electron transport under stress conditions [20]. Moreover, in cv. Mosa, *Xcc*-responsive upregulation of SOD was much higher than that of CAT (Figure 5). This suggests that the H_2_O_2_ produced by SOD-catalyzed dismutation could not be sufficiently scavenged by CAT, thus resulting in higher accumulation of H_2_O_2_.

ROS accumulation induces protein degradation in chloroplasts with their strong photooxidative potential as a hypersensitive response to abiotic and/or biotic stresses [21]. Ribulose-1,5-bisphosphate carboxylase/oxygenase (Rubisco) is the most abundant chloroplast protein [21], and is degraded under stress conditions [22]. *Xcc*-responsive Rubisco subunits (large and small) had a much lower abundance in cv. Mosa than in cv. Capitol (Figure 4), reflecting higher Rubisco degradation in cv. Mosa as a susceptible interaction. Consistently, proteolysis-related proteins, including protease complex, three proteasome subunits, and ATP-dependent Clp protease proteolytic subunits (ClpP), were upregulated only in cv. Mosa (Figure 3). The enzyme ClpP is a highly conserved serine protease present throughout the Bacterial kingdom and found in mitochondria [23], and also in the chloroplasts of eukaryotic cells [24]. The Clp protease system is a central component of the chloroplast protease network [24], responsible for the degradation of numerous stromal proteins. Given that the process of protein degradation is initiated by ROS and involves the action of proteolytic enzymes such as cysteine and serine proteases [21], *Xcc*-responsive enhancement of proteolysis-related proteins in cv. Mosa (Figure 3), which was accompanied by H_2_O_2_ accumulation (Figure 1c), might be part of the symptoms of black rot diseases development in the susceptible cultivar.

### 4.2. Photosynthesis-Related Proteins

Almost all the chemical processes that constitute the light reactions of photosynthesis are carried out by four major protein complexes: photosystem II (PS II), cytochrome b_6_f complex, photosystem I (PS I), and ATP synthase. PS II proteins were highly expressed (especially 22 kDa) in cv. Mosa, and downregulated in cv. Capitol. Red light absorbed by PS II produces a very strong oxidant and a weaker reductant, relative to those produced by PS I [25]. A distinct enhancement of chlorophyll a/b-binding proteins, which are the apoproteins of the light-harvesting complex of PS II [26], was also observed (Figure 4). However, most PS II proteins were expressed or downregulated by *Xcc* infection in cv. Capitol (Figure 4). Light-harvesting complex proteins serve as the antenna complex of chlorophyll and xanthophyll. These antenna complexes absorb sunlight and transfer the excitation energy to the core complexes of PS II in order to drive photosynthetic electron transport [27,28].

In the resistant cultivar (cv. Capitol), a higher enhancement of oxygen-evolving enhancer 3 chloroplastic protein was observed, which releases protons into the lumen [25]. In addition, an increase in the cytochrome *b_6_f* complex Fe-S, which oxidizes plastohydroquinone (PQH_2_) molecules and delivers electrons to PS I [29], was observed. Moreover, in the cv. Capitol, PS I reaction center subunits II, IV, and VI, as well as chlorophyll a/b binding protein in PS I were upregulated (Figure 4) but were lower in abundance or not differentially expressed in cv. Mosa. PS I reduces NAD (P)^+^ to NADPH in the stroma by the action of ferredoxin and the flavoprotein ferredoxin-NADP^+^ reductase [30]. These results suggest that the protein complexes in PS I, which produce a strong reductant and are capable of reducing NAD(P)^+^, were highly expressed in the resistant cultivar, whereas those of PS II were expressed in the susceptible cultivar. The PS I reaction center and its associated antenna pigments, as well as the ATP synthase that catalyzes the formation of ATP, are found exclusively in the stroma lamellae and at the edge of the grana stack [31]. Consistently, the ATP synthase subunit was more highly expressed in cv. Capitol than in cv. Mosa (Figure 4). Therefore, it is noteworthy that the maintenance and/or increased activation of PS I protein complexes might be part of the resistant interaction in the *B. napus–Xcc* pathosystem. Moreover, higher expression of Calvin cycle enzymes, including ribulose biphosphate oxygenase and ribulose-1,5-bisphosphate carboxylase oxygenase large subunit, was remarked in cv. Capitol (Figure 4). Similar results were obtained in the interaction between resistant *B. carinata* and *Leptosphheria maculans* [32], and resistant *B. oleracea* with *Xcc* [11].

### 4.3. Redox-Related Proteins

Oxidation and reduction refer to the transfer of one or more electrons from a donor to an acceptor, usually to another chemical species. These reactions play a central role in the processes of photosynthesis and respiration. In cv. Mosa, mitochondrial pyruvate dehydrogenase, which forms the entry point to the citric acid cycle, was remarkably upregulated, along with malate dehydrogenase, which was downregulated in cv. Capitol (Figure 5). The activation of pyruvate dehydrogenase could be signaled through a decrease in matrix-derived NADH, which enhances the activity of citric acid cycle enzymes such as malate dehydrogenase [33]. This suggests that the electron transport chain operates at an activated rate as the cell’s demand for ATP in the cytosol increases, relative to the rate of ATP synthesis in the mitochondria. Moreover, in cv. Mosa, glycerate dehydrogenase, which functions in the Calvin cycle through the C_2_ oxidative cycle, and geranylgeranyl diphosphate, which is involved in terpene biosynthesis, were highly expressed, but not differentially expressed by *Xcc* infection in cv. Capitol. The fact that geranylgeranyl diphosphate, a precursor of di- and tetraterpene biosynthesis, is formed from the methylerythritol phosphate (MEP) pathway in which the intermediates of glycolysis are involved [34], suggests that higher activation of the C_2_ oxidative cycle and glycolysis could be part of the susceptible interaction with *Xcc*.

In the resistant cultivar (cv. Capitol), *Xcc* infection upregulated 2-cys peroxiredoxin and thioredoxin chloroplastic proteins, whereas no differential expression of these proteins was observed in cv. Mosa (Figure 5). The enzyme 2-Cys peroxiredoxin is involved in scavenging H_2_O_2_ in chloroplasts as part of the ascorbate-glutathione cycle. The levels of *Cu/Zn-SOD* and monodehydroascorbate reductase (*MDHAR*) transcripts and proteins were higher in the tomato 2-cys peroxiredoxin-deficient mutant [35]. Antisense suppression of 2-Cys peroxiredoxin enhanced the activity and expression of *MDHAR* in Arabidopsis [36]. Therefore, our data clearly suggest that the enhanced 2-cys peroxiredoxin (PRX) in cv. Capitol would be associated with lower accumulation of H_2_O_2_ (Figure 1b), SOD (Figure 5), and MDHAR (Figure 5), which were not differentially expressed by *Xcc* infection in a resistant interaction. The observations were the opposite in cv. Mosa in which 2-cys PRX accumulation was absent.

Thioredoxins (TRXs) control many enzymes by reversible redox dimerization of cysteine residues [37]. In the present study, *Xcc* infection-responsive TRX was enhanced only in cv. Capitol. Glutathione S-transferase catalyzes the nucleophilic attack of the sulfur atom of the tripeptide glutathione on electrophilic centers of low-molecular-weight compounds [38], and has been identified as stress response proteins that accumulate in response to biotic and abiotic stimuli. This enzyme was upregulated only in cv. Capitol. Inosine-5′-monophosphate dehydrogenase (IMPDH) is a purine biosynthetic enzyme that catalyzes the nicotinamide adenine dinucleotide (NAD^+^)-dependent oxidation of inosine monophosphate (IMP) to xanthosine monophosphate (XMP). It involves a fast redox reaction involving a hybrid transfer to generate NADH, with a conserved cysteine residue attacking the 2-position of the purine ring [39]. The IMPDH enzyme was upregulated only in the resistant cultivar (Figure 5). Indeed, chemical analysis of NADH-, glutathione (GSH)-, and ascorbate (AsA)-based redox statuses clearly confirmed that reducing potential was significantly enhanced in cv. Capitol, which decreased in cv. Mosa (Figure 7). Previously, we showed that the downregulation of GSH and NADPH-dependent redox status is associated with the susceptibility of *B. napus* cultivars to *Xcc* [2]. Finity et al. [40] reported that the enhancement of GSH- and AsA-dependent redox statuses associated with the hexanoic acid primed defense responses in the tomato against *Botrytis cinerea*. These results indicate that, during a resistant interaction, the TRX- and 2 Cys-PRX-dependent redox signaling and the enhanced reducing potential (NADH/NAD^+^, GSH/GSSG, and AsA/DHA) are the key defense responses to maintain redox homeostasis, thus alleviating the oxidative stress caused by *Xcc* inoculation.

### 4.4. Immune-Responsive Proteins

The plant immune system involves both pattern-triggered immunity (PTI) and effector-triggered immunity (ETI). ETI, which is the long-lasting defense response, has been known to be activated by the interactions between pathogen effector and plant resistance proteins (R-proteins) [41]. R-proteins are usually nucleotide binding-site leucine-rich repeat (NBS-LRR) proteins. Two subfamilies of these proteins can be distinguished: Toll/Interleukin 1 receptor-NBS-LRR (TIR-NBS-LRR) proteins containing the TIR domain and non-TIR-NBS-LRR proteins containing other domains, including the coiled-coil (CC) domain (CC-NBS-LRR) and BED proteins containing a zinc-finger DNA-binding domain [42]. Functional classification of the differentially expressed proteins revealed that the zinc finger SWIM domain-containing 7 isoform X2 (ZFD) protein was upregulated only in cv. Capitol (Figure 6). Moreover, the expression of *ZFD* transcript was highly enhanced by *Xcc* infection in cv. Capitol, but significantly repressed in cv. Mosa (Figure 8a). It has recently been reported that noncanonical N-terminal zinc-finger BED domain-containing NBS-LRR proteins are involved in resistance against the wheat yellow (stripe) rust fungus *Puccinia striiformis* f. sp. *tritici* [43].

RNA-binding proteins (RBPs) play an important role in the post-transcriptional modification of RNA, and are induced by biotic and abiotic stress [44]. In the present study, we found that glycine-rich RNA-binding GRP1A isoform X1 (GRP1A) protein was upregulated only in cv. Capitol (Figure 6), consistent with enhancement of its gene (*GRP*) expression in cv. Capitol, relative to that in cv. Mosa (Figure 8b). Arabidopsis mutant lacking *GRP7* has been found to be more susceptible to bacterial speck disease caused by *Pseudomonas syringae* pv. *tomato* [41]. ETI, which constitutes mainly programmed cell death (PCD), is induced by diverse mechanisms, including mitochondrial permeability transition (MPT), phospholipase Ds (PLDs), and PLD-derived phosphatidic acid (PA) signaling [45,46]. MPT is induced by the mitochondrial outer membrane porins [45]. In this study, we found that the mitochondrial outer membrane porin-3-like protein was highly upregulated in cv. Capitol, whereas phospholipase D alpha 2 was downregulated in cv. Mosa in response to *Xcc* inoculation (Figure 6). Swidzinski et al. [47] reported that mitochondrial outer membrane porin accumulation was induced during senescence or heat shock-induced PCD as a hypersensitive response. These data suggest that the R-proteins containing ZFD and GRP1A would be key regulators of ETI; alternatively, R protein-mediated activation of MPT through the enhanced accumulation of mitochondrial outer membrane porin triggers PCD, thus inducing resistance in the *B. napus–Xcc* pathosystem.

## 5. Conclusions

In conclusion, these results indicate that induction of H_2_O_2_ as oxidative stress and proteolysis-related protein accumulation occurred in susceptible interactions. Higher activation of PS I is involved in resistance, whereas maintenance of PS II is associated with susceptibility to *Xcc*. Enhanced mitochondrial permeability transition induces PCD and the upregulation of the redox signaling proteins, along with the higher reducing potential, were key resistance responses in the *B. napus–Xcc* pathosystem. Taken together, this study reveals complex innate defense mechanisms and suggests important molecular targets that have broad potential implications in future metabolic engineering or breeding approaches to develop resistant crop cultivars to *Xcc*.

## Data Availability

Data is contained within the article or supplementary material.

## Supplementary Materials

The following are available online at https://www.mdpi.com/2076-2607/9/2/253/s1, Figure S1: Venn diagram illustrating the number of identified proteins in resistant (cv. Capitol) and susceptible (cv. Mosa) cultivars of *B. napus* in responses to *Xcc*, Figure S2: Functional classification of *B. napus* resistant cultivar (cv. Capitol) proteins in responses to *Xcc*; Proteins were classified by gene ontology analysis with BLAST2GO on the basis of their cellular component, Figure S3: Functional classification of *B. napus* susceptible cultivar (cv. Mosa) proteins in responses to *Xcc*; Proteins were classified by gene ontology analysis with BLAST2GO on the basis of their molecular function, Table S1: Specific primers used for qRT-PCR., Table S2: Annotation of differentially accumulated proteins using Blast2GO in *B. napus* resistant cultivar (cv. Capitol) in responses to *Xcc*, Table S3: Annotation of differentially accumulated proteins using Blast2GO in *B. napus* susceptible cultivar (cv. Mosa) in responses to *Xcc*.
