# Supplementary material for: Label-Free Quantitative Proteomics Analysis in Susceptible and Resistant Brassica napus Cultivars Infected with Xanthomonas campestris pv. campestris
Source: Microorganisms. 2021 Jan 27;9(2):253. doi: 10.3390/microorganisms9020253 (PMC7911590; doi:10.3390/microorganisms9020253)
Supplement: Supplementary file 1 [file microorganisms-09-00253-s001.zip › Suplementary Table S3.docx]

**Supplementary Table S3**. Specific primers used for qRT-PCR.

| **Target gene** | **GenBank Number** | **Forward sequence** | **Reverse sequence** |
| --- | --- | --- | --- |
| ZFD | XM_013885309.2 | 5´- ACCTTGAGAAAGCGACCAGA - 3´ | 5´- GCAGCCAATTGATGCTTACA - 3´ |
| GRP | XM_013805486.1 | 5´- TCCCCCTCTCTCTCATTTCA - 3´ | 5´- GCGTCCTTCATCGACTTCTC - 3´ |
| 2-Cys-PRX | AF311863.1 | 5´- CCCTCAAAGCCTTCTCCTTT - 3´ | 5´- AAAAACAGCCTCTGCCTCAA - 3´ |
| TRX | XM013811982 | 5´- GAAGTCTGGAGCGAGAAGAT - 3´ | 5´- CGATCTTGAAGAAGACAACG - 3´ |
| ACTIN | AF111812 | 5´- GATTCCGTTGCCCTGAAGTA - 3´ | 5´- GCGACCACCTTGATCTTCAT- 3´ |

All primers were designed directly from sequences in the public database.
