# Supplementary material for: Label-Free Quantitative Proteomics Analysis in Susceptible and Resistant Brassica napus Cultivars Infected with Xanthomonas campestris pv. campestris
Source: Microorganisms. 2021 Jan 27;9(2):253. doi: 10.3390/microorganisms9020253 (PMC7911590; doi:10.3390/microorganisms9020253)
Supplement: Supplementary file 1 [file microorganisms-09-00253-s001.zip › Supplementary Figures.pptx]

## Slide 1
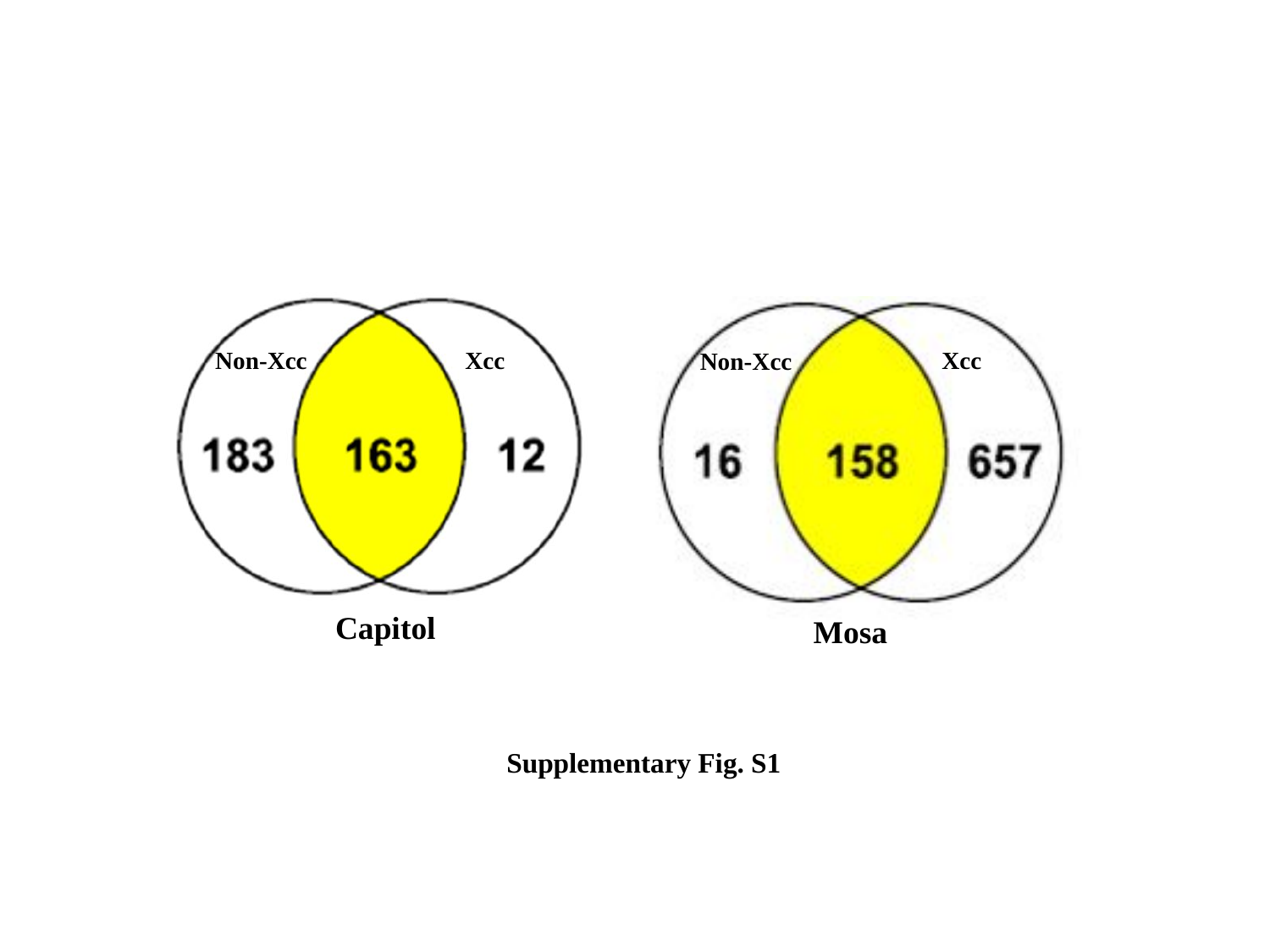

Xcc
Xcc
Non-Xcc
Non-Xcc
Capitol
Mosa
Supplementary Fig. S1

## Slide 2
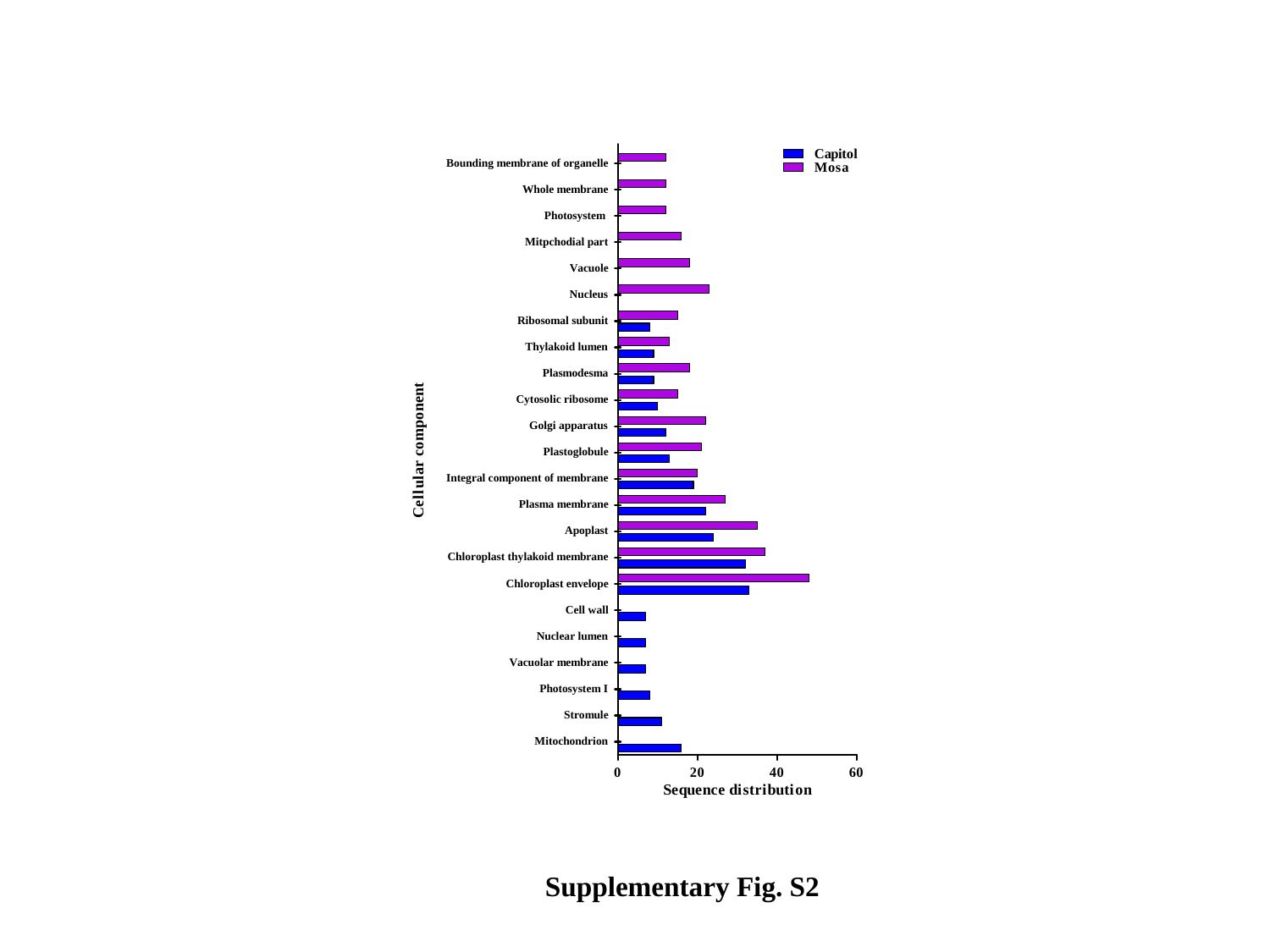

Supplementary Fig. S2

## Slide 3
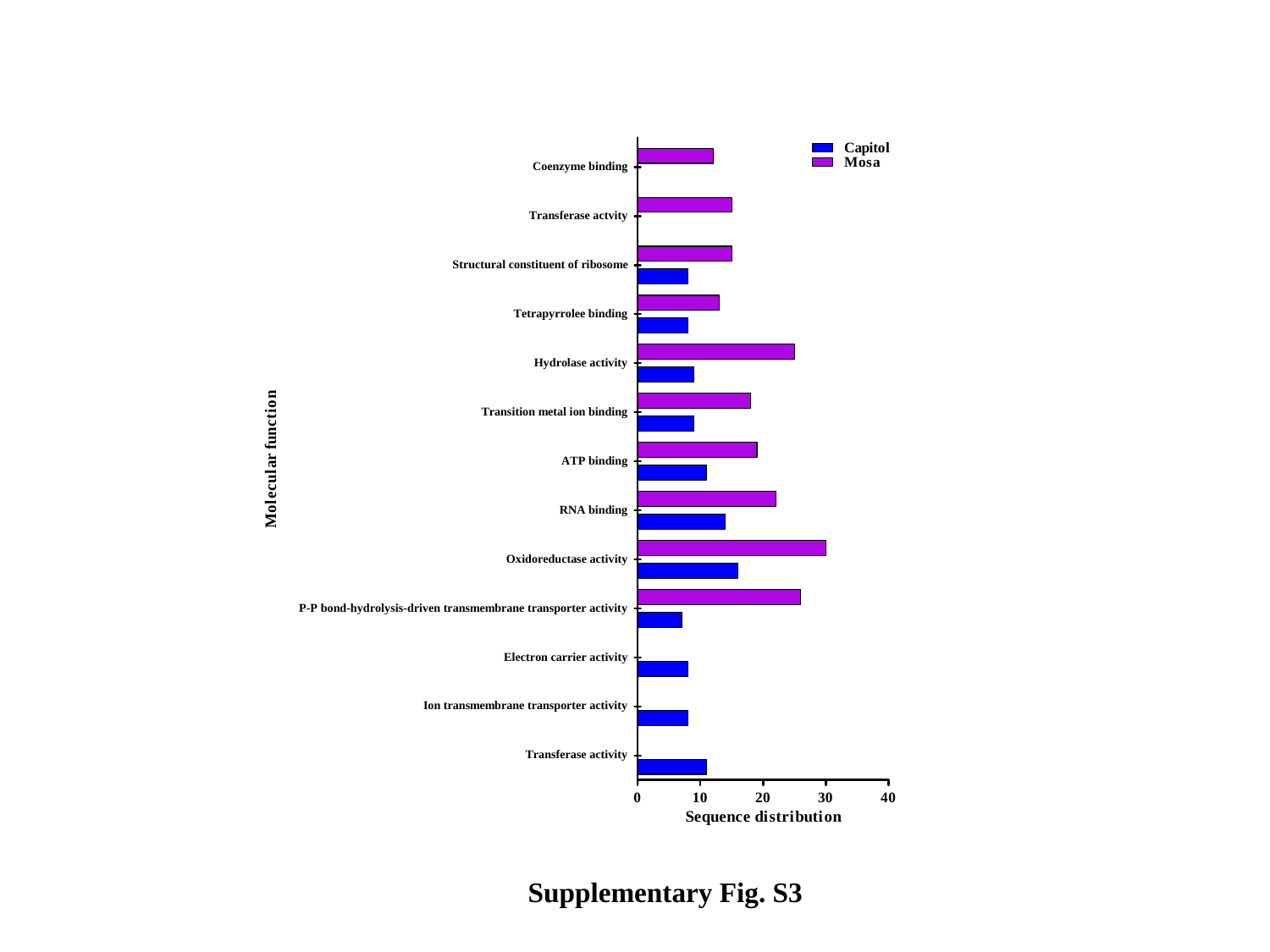

Supplementary Fig. S3
